# Oral Health Knowledge and Habits of People With Type 1 and Type 2 Diabetes

**DOI:** 10.1016/j.identj.2021.07.003

**Published:** 2021-09-08

**Authors:** Dorottya Banyai, Adam Vegh, Zita Biczo, Mark Thomaz Ugliara Barone, Tamás Hegedus, Daniel Vegh

**Affiliations:** a Department of Pedodontics and Orthodontics, Semmelweis University, Budapest, Hungary; b Diabetes-Dental Working Group, Semmelweis University, Budapest, Hungary; c Department of Maxillofacial and Oral Surgery, Semmelweis University, Budapest, Hungary; d Faculty of Dentistry, Dental Student, Semmelweis University, Budapest, Hungary; e International Diabetes Federation, Brussels, Belgium; f ADJ Diabetes Brasil, São Paulo, Brazil; g Fórum Intersetorial para Combate às DCNTs no Brasil, São Paulo, Brazil; h Department of Prosthodontics, Semmelweis University, Budapest, Hungary

**Keywords:** Diabetes, Oral health, Advocacy, Type 1 diabetes, Health education, Public health

## Abstract

**Objectives:**

This study aimed to collect information about oral health knowledge and the habits of people living with diabetes (PwD), primarily type 1 diabetes, using the newly developed World Health Organisation Oral Health Questionnaire for Adults (Annex 7).

**Materials and methods:**

Comparable and reliable questionnaires, comprising 23 questions for PwD, were sent to diabetes social media groups, mailing lists, and associations. The survey explored the relationships amongst demographic factors, age, dental education, eating habits, and other factors.

**Results:**

The 23-question survey was answered by 307 individuals from 60 different countries. Alcohol and tobacco use, dental anxiety, and bad habits were often reported. Of the participants, 61.2% (n = 188) had at least 1 drink during the past 30 days. Of the participants, 22.8% (n = 70) were smokers. In total, 80.8% (n = 248) of the participants consumed biscuits, 76.2% (n = 234) consumed sweets, and 63.2% (n = 194) consumed soft drinks regularly. A total of 26.4% (n = 81) of the participants reported being afraid of dental treatment. Of the participants, 48.5% (n = 149) reported dry mouth and other oral complications. The frequency of visits to the dentist was satisfactory. A total of 71.3% (n = 219) of the participants reported visiting a dentist during the past 12 months.

**Conclusions:**

There is a need for proper oral health education for PwD. Trained diabetes advocates could be core messengers. However, interdisciplinary cooperation is mandatory for both education and the clinical aspect of diabetes care. For example, diabetes nurses need to be educated with the help of dentists or oral hygienists.

## Introduction

Diabetes mellitus is a chronic metabolic disease characterised by elevated blood glucose levels, which is caused by a lack of insulin secretion, function, or both.^1^ There are 3 main types of diabetes: type 1 diabetes mellitus (T1D), type 2 diabetes mellitus (T2D), and gestational diabetes mellitus, along with unique subtypes.^2^ The pathophysiology of T1D is based on an autoimmune reaction against insulin-producing β-cells of the pancreas.^2^ Unfortunately, it is still uncertain what initiates this process; however, genetic susceptibility and environmental triggers, such as viral infections, are suspected.^2^ T2D is more likely associated with physical inactivity and poor diet, which leads to obesity.^2^ Therefore, T2D could be prevented relatively easily with behavioural interventions focusing on risk factors. Despite this, T2D is the most common form of diabetes and represents a significant public health problem worldwide. In 2017, there were 451 million people living with diabetes (PwD) worldwide, particularly in low- or middle-income countries. This prevalence is expected to increase to 693 million by 2045.^3^ Poor glycemic control can lead to serious, potentially life-threatening complications, such as critical limb ischemia, foot infections requiring amputation,^4^ kidney failure,^5^ blindness,^6^ or cardiovascular diseases.^7^ In addition to many systemic adverse effects that are correlated with diabetes, there may also be a high prevalence of oral complications, including xerostomia, delayed wound healing, increased formation of oral cancers,^8^ taste impairment, oral candidiasis, oral lichen planus,^9^ and periodontal disease (PD).^10^ Patients with poor or uncontrolled glycemia are more susceptible to PD.^11^^,^^12^ Additionally, in a vicious circle, periodontal infection may adversely affect metabolic control of diabetes.^13^^,^^14^

Unsatisfactory glycemic control (glycosylated hemoglobin [HbA1c] >7%) and chronic hyperglycemia correlate with clinical attachment loss and periodontal tissue destruction.^15^ Tooth loss associated with PD is usually higher in patients with T1D because of the more prolonged course of this metabolic disease.^15^

In T1D, elevated levels of inflammatory cytokines (prostaglandin E_2_ and interleukin-1β) correlated with increasing HbA1c values. Cellular dysfuction affecting polymorphonuclear leukocytes, cell chemotaxis, and apoptosis increase their retention in the periodontal tissues and cause more destruction. In addition, the elevated advanced glycation end products/receptors for advanced glycation end products interaction upregulates the production of inflammatory mediators and the more intense tumor necrosis factor-α reaction to periodontal pathogens such as *Porphyromonas gingivalis*.^16^

Hyperglycemia has an upregulating effect on receptor activator of nuclear factor-kappa B ligand, leading to osteoclast activation and more severe bone resorption.^17^ Severe PD has local and systemic harmful metabolic effects, thereby completing the vicious circle.^17^

This study aimed to collect information about the oral health knowledge and habits of PwD. Our main objective was to understand the habits and oral health education level in PwD. Most international studies have focused on the population with T2D. The number of T2D cases accounts for 90% to 95% of the population with diabetes, making data collection much more accessible. Therefore, we aimed to assess the overall oral health and oral habits of people living with T1D (PwT1D).

## Material and methods

### Participants

Participants belonging to the International Diabetes Federation's (IDF's) Young Leaders in Diabetes (YLD) Programme were asked to share the questionnaire online with their local diabetes community members, along with the help and approval of their local diabetes associations. People could answer the questionnaire between December 15, 2019, and January 31, 2020, using a Google Survey form, accessible on a computer, tablet, or cell phone.

Participation in the questionnaire survey was voluntary. Exclusion criteria included an incomplete questionnaire and more than one sample from the same Internet Protocol or email address (possible duplicate answer). People without diabetes were excluded from the study. The questionnaire was in English; however, the auto-translation mode to Spanish was available, which might have limited the participation of other language speakers.

### Online survey

The study protocol was based on a World Health Organisation (WHO) survey.^18^ This survey was "pilot-tested" in a range of countries across the world. These simplified questionnaires include core questions that are considered essential in national oral health surveillance. We recorded the following information: sex, age, country of origin, location, type of diabetes, number of teeth, any discomfort in the oral cavity, removable dentures, oral care habits, oral health status, oral care equipment use, toothpaste type used, frequency of dental visits, food and alcohol consumption, smoking habits, primary education, and possible fear of dental treatment. The study was approved by the Semmelweis University Ethical Board and was conducted in accordance with the Declaration of Helsinki.

### Data collection and outcome measures

We collected the data online. All data were stored using Google Survey and Microsoft Excel.

### Statistical analysis and visualisation

Data analysis was performed using Prism version 8.4.2. (Graphpad Software) software, and data are reported as means ± standard deviations (SDs) and range or absolute numbers with percentages. We used Pearson's Chi-squared test for statistical analysis. Differences below the 5% limit (*P* < .05) were considered significant. For visualisation, we used Tableau Public (Tableau Software).

## Results

A total of 307 patients, comprising 89 men and 218 women from 60 different countries worldwide, participated in this survey (Figure).

**Fig. 1:**
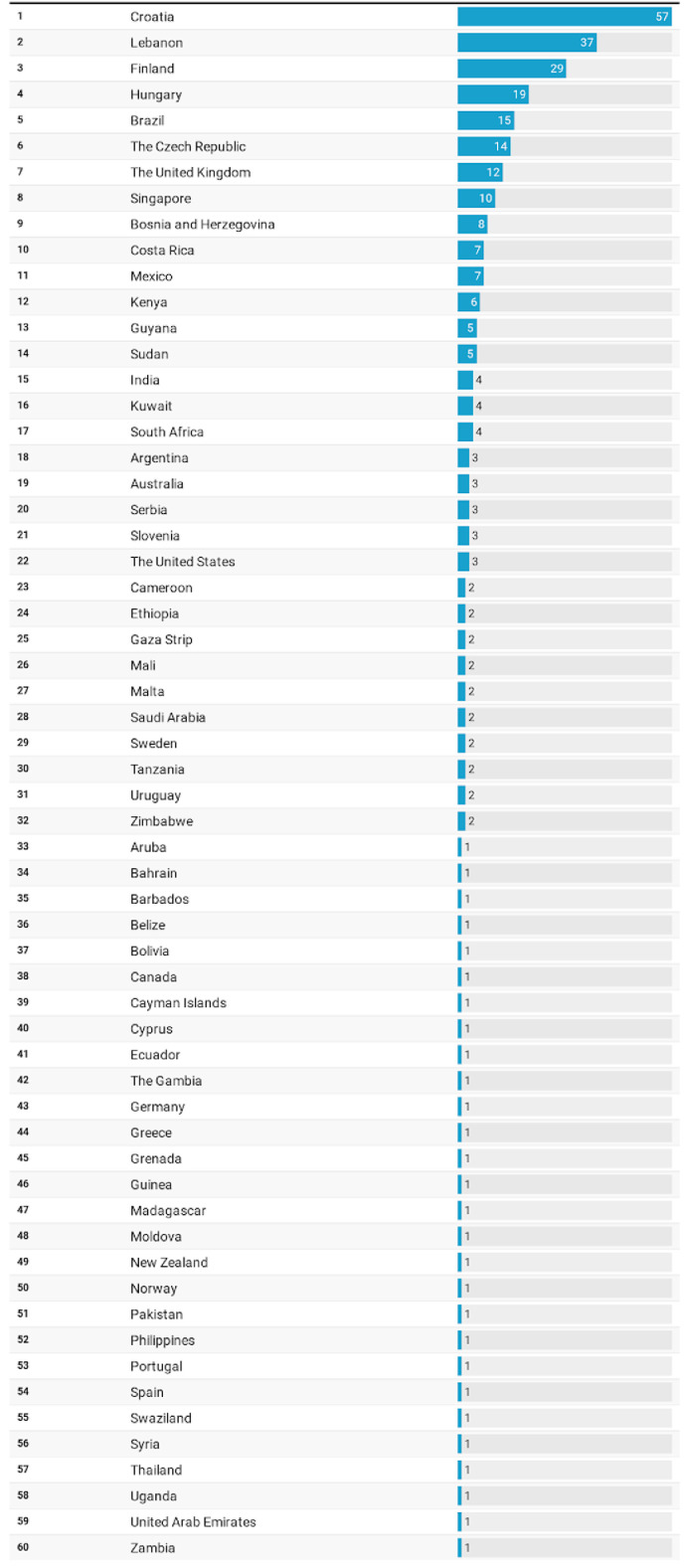
Respondents by country of origin.

With respect to the global regions, responses were received from the following: South and Central America, 10.1% (n = 31) of the participants; North America and the Caribbean, 6.8% (n = 21) of the participants; Europe, 50.8% (n = 156) of the participants; Africa, 8.8% (n = 27) of the participants; Southeast Asia, 1.6% (n = 5) of the participants; Western Pacific, 5.6% (n = 17) of the participants; and the Middle East and North Africa, 16.3% (n = 50) of the participants. The most responsive countries, comprising 40% of the answers, were Croatia, 18.6% (n = 57); Lebanon, 12.1% (n = 37); Finland, 9.4% (n = 29); Hungary, 6.2% (n = 19); and Brazil, 4.9% (n = 15). A total of 73.6% (n = 226) of the feedback was from participants living in urban areas (Table 1).

**Table 1:** Sociodemographic characteristics.

		n (%)		
	Female	Male	Total	*P* value
Overall	218 (71.0)	89 (29.0)	307 (100.0)	
Region				.7408
Europe	103 (66.0)	53 (34.0)	156 (50.8)	
Middle East and North Africa	36 (72.0)	14 (28.0)	50 (16.3)	
South and Central America	23 (74.2)	8 (25.8)	31 (10.1)	
Africa	20 (74.1)	7 (25.9)	27 (8.8)	
North America and the Caribbean	17 (81.0)	4 (19.0)	21 (6.8)	
Western Pacific	13 (76.5)	4 (23.5)	17 (5.6)	
Southeast Asia	4 (80.0)	1 (20.0)	5 (1.6)	
Area				.8843
Rural	27 (73.0)	10 (27.0)	37 (12.1)	
Periurban	30 (68.2)	14 (31.8)	44 (14.3)	
Urban	161 (71.2)	65 (28.8)	226 (73.6)	
Education level				.7827
College/university completed	98 (73.1)	36 (26.9)	134 (43.6)	
High school completed	55 (64.7)	30 (35.3)	85 (27.7)	
Postgraduate degree	40 (74.1)	14 (25.9)	54 (17.6)	
Secondary school completed	19 (73.1)	7 (26.9)	26 (8.5)	
Less than primary school	2 (66.7)	1 (33.3)	3 (1.0)	
No formal schooling	4 (80.0)	1 (20.0)	5 (1.6)	

Of the participants, 47.9% (n = 147) were aged between 20 and 30 years. The mean age was 30.4 years (SD = ±12.4; range 2-72 years). PwD who were younger than 18 years could only participate in the survey with parental surveillance/help. The participants were well educated, as 43.6% (n = 134) had a college or university degree and 17.6% (n = 54) had a postgraduate degree. Altogether, 97.4% (n = 299) of the attendees had a high school education or higher education level. The online form of the questionnaire indicated that most participants are well educated, are living in urban areas, and are health conscious. All 307 participants had diagnosed diabetes: 85% (n = 261) of the participants had T1D and 15% (n = 46) had T2D. Of the respondents, 91.2% (n = 280) reported having 20 teeth or more, and 4.6% (n = 14) claimed to have 10 to 19 natural teeth. Of the participants, 1.3% (n = 4) had 1 to 9 natural teeth. Amongst the participants, 8.1% (n = 25) had a partial denture, and 2.9% (n = 9) reported that they were completely edentulous. Of the participants, 2% (n = 6) wore a complete upper denture, 2.6% (n = 8) had a complete lower denture, and 1% (n = 3) used both complete upper and lower dentures. Approximately half of the participants, 45.3% (n = 139), experienced discomfort or pain associated with their teeth or mouth during the previous 12 months. Of these patients, 46.9% (n = 144) visited their dentist in the last 6 months. Almost one-third of the participants, 28.7% (n=88), did not visit a dentist in the last 12 months, even when they experienced pain. Of the participants, 48.5% (n = 149) complained of dry mouth, and 32.2% (n = 99) of all answers, with women accounting for 74.7% (n = 74) of these answers, claimed that they were embarrassed due to the appearance of their teeth. Based on these answers, appearance posed a bigger problem for patients rather than mastication. In total, 28.7% (n = 88) of the participants experienced difficulty in mastication. In the entire group, the rate of dental anxiety was 26.4% (n = 81). We found a significant gender difference in dental anxiety due to this self-reported survey (*P* = .000367, *P* < .05). Women (86.4%, n = 70) were more likely to fear dentists or dental treatment than men (13.6%, n = 11).

Despite this, most respondents, 81.4% (n = 250), described the status of their teeth and gums as "average," "good," or "very good." Based on the survey, 71.3% (n = 219) of the participants visited a dentist in the last 12 months, most of them even in the last 6 months.

Half of those who visited a dentist in the last 6 months only needed a routine checkup. Two-thirds of the participants scheduled a dentist appointment as a routine checkup and treatment, if needed, or for a follow-up treatment. A total of 22.5% (n = 69) of the participants visited a dentist due to pain or discomfort associated with their teeth, gums, or mouth. Of the participants, 8.5% (n = 26) had only a consultation with their dentist. Unfortunately, 2.3% (n = 7) of PwT1D never received dental care. As part of the home hygiene routine, 99% (n = 304) of the participants used a toothbrush, 29% (n = 89) used a wooden toothpick, 21.2% (n = 65) used a plastic toothpick, 60.3% (n = 185) used dental floss, 8.5% (n = 26) used charcoal, 7.2% (n = 22) used chewstick/miswak, 15.6% (n = 48) used an interdental brush, and 19.5% (n = 60) used other types of oral health equipment. In a survey conducted in the US, the frequency of floss usage was 33%.^19^

We observed that 71.3% (n = 219) of the participants brushed their teeth twice or more a day, and 24.8% (n = 76) performed this routine once a day. Electric toothbrushes were not widely used amongst our participants. In total, 13.7% (n = 42) of the participants used only an electric toothbrush. Of the participants, 11.4% (n = 35) claimed to use both ordinary and electric toothbrushes. Only 67% (n = 175) of the participants used fluoride-containing toothpaste, and this proportion amongst people living with T2D (PwT2D) was 83% (n = 38). It should be mentioned that 20.5% (n = 63) of all respondents did not know whether their toothpaste contained fluoride. Dental floss was the most preferred interdental cleaning product. A total of 60.3% (n = 185) of the participants reported flossing regularly, whilst only 15.6% (n = 48) chose interdental brushes. A total of 12% (n = 37) of the participants reported using both dental floss and an interdental brush. Our survey also demonstrated that 53.1% (n = 163) of the participants had prior oral care education. Unfortunately, approximately half of the participants had never received education on oral care protocols. In the last section, we attempted to determine the bad habits of the respondents (Table 2).

**Table 2:** Dental status and compliance.

		n (%)		
	Female	Male	Total	*P* value
Type of diabetes				.5574
Type 1 diabetes mellitus	187 (71.6)	74 (28.4)	261 (85.0)	
Type 2 diabetes mellitus	31 (67.4)	15 (32.6)	46 (15.0)	
Number of teeth				.9701
≥20 teeth	198 (70.7)	82 (29.3)	280 (91.2)	
10-19 teeth	10 (71.4)	4 (28.6)	14 (4.6)	
1-9 teeth	3 (75.0)	1 (25.0)	4 (1.3)	
No natural teeth	7 (77.8)	2 (22.2)	9 (2.9)	
Removable dentures	18 (72.0)	7 (28.0)	25 (8.1)	.9094
Full upper denture	6 (100.0)	0 (0.0)	6 (2.0)	.8794
Full lower denture	6 (75.0)	2 (25.0)	8 (2.6)	.8010
Experienced trouble during the past 12 months	106 (76.3)	33 (23.7)	139 (45.3)	.5450
Difficulty biting or chewing food	65 (73.9)	23 (26.1)	88 (28.7)	
Difficulty with speech	36 (63.2)	21 (36.8)	57 (18.6)	
Dry mouth	110 (73.8)	39 (26.2)	149 (48.5)	
Felt embarrassed due to appearance of teeth	74 (74.7)	25 (25.3)	99 (32.2)	
Reduced participation in social activities	40 (70.2)	17 (29.8)	57 (18.6)	
Time since the last dental visit				.4233
<6 months	103 (71.5)	41 (28.5)	144 (46.9)	
6-12 months	58 (77.3)	17 (22.7)	75 (24.4)	
>1 year but <2 years	30 (71.4)	12 (28.6)	42 (13.7)	
≥2 years but <5 years	15 (57.7)	11 (42.3)	26 (8.5)	
≥5 years	8 (61.5)	5 (38.5)	13 (4.2)	
Never received dental care	4 (57.1)	3 (42.9)	7 (2.3)	
Reason of the last visit				.8433
Routine checkup/treatment	97 (69.8)	42 (30.2)	139 (45.3)	
Treatment/follow-up treatment	43 (71.7)	17 (28.3)	60 (19.5)	
Pain or trouble with teeth, gums, or mouth	48 (69.6)	21 (30.4)	69 (22.5)	
Don't know/don't remember	11 (84.6)	2 (15.4)	13 (4.2)	
Consultation/advice	19 (73.1)	7 (26.9)	26 (8.5)	
Afraid of dental treatment	70 (86.4)	11 (13.6)	81 (26.4)	.000367

A total of 94.8% of the participants claimed to eat fresh fruits daily. However, 80.8% (n = 248) of the participants also claimed to eat biscuits and cakes. Sweets and candies were preferred as much as biscuits and cakes (76.2%, n = 234). Of the PwT1D, 48% (n = 125) claimed to eat candies. Of the PwT1D, 31% (n = 81) drank lemonade or cola weekly or more often. Tea (29.3%, n = 90) or coffee with sugar (26.4%, n = 81) was less preferred. A total of 77.2% (n = 237) of the participants stated that they never smoked cigarettes (in the US survey, 51.1%^19^); however, 20 participants, 8% of PwT1D, admitted that they smoked every day (the US survey, 19%^19^). Considering the national diversity of the participants, other tobacco types (cigars, pipe, snuff, or tobacco chewing) were also consumed; however, they were not preferred and were consumed occasionally. Of the participants, 38.8% (n = 119) said they did not consume alcohol during the past 30 days. A total of 44.3% (n = 136) of the participants claimed to have <1, 1, or 2 drinks. A total of 3.9% (n = 12) of the respondents admitted having ≥5 drinks in the last month. In the US survey, 47.9% had ≥4 drinks in the previous month (Table 3).

**Table 3:** Oral hygiene and habits.

		n (%)		
	Female	Male	Total	*P* value
Reported frequency of tooth brushing				.0833
Twice or more a day	165 (75.3)	54 (24.7)	219 (71.3)	
Once a day	48 (63.2)	28 (36.8)	76 (24.8)	
2-6 times a week	4 (44.4)	5 (55.6)	9 (2.9)	
2-3 times a month	1 (50.0)	1 (50.0)	2 (0.7)	
Once a month	1 (100.0)	0 (0.0)	1 (0.3)	
Used dental cleaning tools				.7354
Toothbrush	216 (71.1)	88 (28.9)	304 (99.0)	.8975
Normal toothbrush	160 (70.5)	67 (29.5)	227 (73.9)	
Electric toothbrush	30 (71.4)	12 (28.6)	42 (13.7)	
Both	26 (74.3)	9 (25.7)	35 (11.4)	
Toothpaste	217 (71.1)	88 (28.9)	305 (99.3)	.5862
Fluoride-containing	153 (71.5)	61 (28.5)	214 (69.7)	
Not fluoride-containing	23 (76.7)	7 (23.3)	30 (9.8)	
Don't know	42 (66.7)	21 (33.3)	63 (20.5)	
Wooden toothpick	62 (69.7)	27 (30.3)	89 (29.0)	
Plastic toothpick	50 (76.9)	15 (23.1)	65 (21.2)	
Dental floss	137 (74.1)	48 (25.9)	185 (60.3)	
Charcoil	21 (80.8)	5 (19.2)	26 (8.5)	
Chewstick/miswak	16 (72.7)	6 (27.3)	22 (7.2)	
Interdental brush	31 (64.6)	17 (35.4)	48 (15.6)	
Other	39 (65.0)	21 (35.0)	60 (19.5)	
Had oral care education	118 (72.4)	45 (27.6)	163 (53.1)	.5699
Eating, drinking habits				.9846
Fresh fruit	207 (95.5)	84 (94.4)	291 (94.8)	
Biscuits	174 (79.8)	74 (75.5)	248 (80.8)	
Chewing gum with sugar	62 (28.4)	32 (36.0)	94 (30.6)	
Sweets/candy	163 (74.8)	71 (79.8)	234 (76.2)	
Soft drinks	136 (62.4)	58 (65.2)	194 (63.2)	
Tea with sugar	63 (28.9)	27 (30.3)	90 (29.3)	
Coffee with sugar	58 (26.6)	23 (25.8)	81 (26.4)	
Drinking alcohol during the past 30 days				.3012
Did not drink alcohol	85 (39.0)	34 (38.2)	119 (38.8)	
<1 drink	37 (17.0)	10 (11.2)	47 (15.3)	
1 drink	31 (14.2)	7 (7.9)	38 (12.4)	
2 drinks	31 (14.2)	20 (22.5)	51 (16.6)	
3 drinks	16 (7.3)	8 (9.0)	24 (7.8)	
4 drink	11 (5.0)	5 (5.6)	16 (5.2)	
≥5 drinks	7 (3.2)	5 (5.6)	12 (3.9)	
Smoking				.7506
Cigarettes	38 (17.4)	17 (19.1)	55 (17.9)	
E-cigarette	11 (5.0)	4 (4.5)	15 (4.9)	

## Discussion

We compared the data presented here with two similar studies. One was conducted in the US^19^ only in PwT1D and another in the UK in respondents having T1D and T2D.^20^ The mean age of our participants was 30.4 years (SD = ±12.4), whilst the mean age of the US T1D attendees (n=390) was 32.6 ± 0.04 years.^19^

Of these respondents, 97.4% were well educated. In comparison, 69% of another T1D survey's^19^ participants had at least a high school education level in the US.

Based on this survey, 71.3% (n = 219) of the participants had annual dental visits, which accounted for 68.9% (n = 268) of the participants with T1D in a US-based study^19^ and 40.4% (n = 194) of the participants with T2D in a UK-based study.^20^

Of these respondents, 71.3% (n = 219) brushed at least twice a day, with similar results in the US T1D survey; 72.2% brushed twice or more frequently.^19^ This value was 67.2% in the UK survey.^20^

Flossing habits revealed greater differences amongst different populations, accounting for 60.3% (n = 185) in this study and 33% (n = 129) amongst PwT1D in the US survey,^19^ and fewer than 50% (n = 63) amongst PwT2D in the UK survey.^20^

Several previous studies have provided evidence that periodontitis is strongly associated with both diabetes and oral hygiene.^9^, ^10^, ^11^, ^12^, ^13^, ^14^ It is also known that interdental cleaning is crucial in maintaining excellent oral hygiene and preventing gingival diseases and PD.^21^^,^^22^ Our survey found that more than half of the people without oral care training never flossed. Even fewer participants used interdental brushes. Unfortunately, PwT1D and PwT2D were found to have limited knowledge about oral care and oral health complications associated with diabetes. Attendance for dental therapy was satisfactory amongst the participants selected for this study (mainly living in urban areas and highly educated). The study revealed that even with regular dental care, the participants in the study needed further education to prevent dental diseases. Therefore, dentists must be educated about diabetes and oral complications in diabetes. Trained diabetes advocates could be core messengers.^23^ However, some people did not receive professional dental care, particularly edentulous individuals.

In general, the prevention of dental diseases remains a challenge. Most people have basic knowledge about the systemic effects of poor glycemic control; however, they need to be educated regarding oral complications and how to prevent them. It was an important moment when experts of the European Federation of Periodontology (EFP) and IDF gathered in Madrid to review the latest findings on the links between PD and diabetes. After this workshop, a consensus guideline was created for physicians, oral health care professionals, and patients to improve early diagnosis, prevention, and co-management of diabetes and periodontitis.^24^ The IDF also produced a guide titled "Oral Health for People With Diabetes," which was an important step. This material emphasises the importance of annual dental checkups and education for people with diabetes, including explaining the implications of diabetes on oral health.^25^ Despite this, it is recommneded that education should also be used to facilitate understanding of the potential or established disease complications. Health coaching is shown to be a promising way to achieve goals in diabetes care. It includes and encourages healthy habits, provides emotional support to cope with the challenges of chronic diseases, and ensures regular follow-up.^26^, ^27^, ^28^

This self-reported study has certain limitations due to the subjective evaluation of the participants’ overall wellness and health status. Nevertheless, this study provides evidence on general oral health awareness and education. Interdisciplinary cooperation is needed amongst dentists, diabetologists, internists, and other health care professionals. Annual dental examinations should be recommended in diabetes care guidelines to improve the lives of PwD.

## Conclusions

PwD see their nurses or family physicians more often than they see their dentists. Therefore, these health care professionals should also be educated with the help of dentists and oral hygienists. In this way, accurate information about oral care can be delivered and patients can be motivated to develop better oral hygiene and avoid harmful habits. The IDF Diabetes Atlas^29^ depicts, and our global survey confirms, that diabetes education varies widely globally, and social awareness regarding diabetes is also remarkably diverse. It must also be mentioned that access to care is unequal across different continents. To diminish this impact, we need to guarantee that oral health is amongst the international priorities of the IDF, WHO, Non-Communicable Disease Alliance, and United Nations Children's Emergency Fund. These survey results showed no significant differences in the habits, socioeconomic factors, or dental status between men and women; however, women were significantly more afraid of dental treatment than men.

The necessity of annual dental checkups cannot be overstated. In particular, PwD are more prone to PD, candidiasis, dry mouth, and dental caries. In addition, poor glycemic control and PD have a back and forth triggering effect, as elevated blood glucose levels make them more prone to infection.

Regular dental examinations are crucial, and the prevalence and therapy for PD have been highlighted in the Madrid consensus by the EFP and IDF.^24^

At the Semmelweis University, Hungary, the Diabetes Dental Working Group provides these essential and free annual dental checkups for PwD, referred by other health care professionals or nongovernmental organisations. Further studies (multiple locations, multicentre) performed by dentists or periodontists are required to monitor the global overview of the oral health status, diabetes status, and oral health knowledge. Interdisciplinary cooperation and oral health promotion should be emphasized in the future by global organisations such as the IDF. Besides health care professionals, diabetes advocates from Blue Circle Voices and YLD programmes could be messengers to reach out to patients and patient groups.

## Conflict of interest

None disclosed.
